# A Novel Lightweight Approach to COVID-19 Diagnostics Based on Chest X-ray Images

**DOI:** 10.3390/jcm11195501

**Published:** 2022-09-20

**Authors:** Agata Giełczyk, Anna Marciniak, Martyna Tarczewska, Sylwester Michal Kloska, Alicja Harmoza, Zbigniew Serafin, Marcin Woźniak

**Affiliations:** 1 Faculty of Telecommunications, Computer Science and Electrical Engineering, Bydgoszcz University of Science and Technology, 85-796 Bydgoszcz, Poland; 2 Faculty of Medicine Ludwik Rydygier Collegium Medicum in Bydgoszcz, Nicolaus Copernicus University in Torun, 85-067 Bydgoszcz, Poland

**Keywords:** features extraction, X-ray images, COVID-19, machine learning, image processing

## Abstract

Background: This paper presents a novel lightweight approach based on machine learning methods supporting COVID-19 diagnostics based on X-ray images. The presented schema offers effective and quick diagnosis of COVID-19. Methods: Real data (X-ray images) from hospital patients were used in this study. All labels, namely those that were COVID-19 positive and negative, were confirmed by a PCR test. Feature extraction was performed using a convolutional neural network, and the subsequent classification of samples used Random Forest, XGBoost, LightGBM and CatBoost. Results: The LightGBM model was the most effective in classifying patients on the basis of features extracted from X-ray images, with an accuracy of 1.00, a precision of 1.00, a recall of 1.00 and an F1-score of 1.00. Conclusion: The proposed schema can potentially be used as a support for radiologists to improve the diagnostic process. The presented approach is efficient and fast. Moreover, it is not excessively complex computationally.

## 1. Introduction

COVID-19 is a disease caused by the SARS-CoV-2 virus. It has a wide range of symptoms, most of which affect the respiratory tract. It can lead to serious inflammation of the lungs and, consequently, pneumonia [1]. The COVID-19 pandemic has exposed healthcare problems around the world. The large number of patients to diagnose and the limited number of tests and staff available turned out to be a significant problem. As a result, the number of diagnostic tests performed was very often too low. The diagnostic method that turned out to be the gold standard for the confirmation of SARS-CoV-2 infection was the polymerase chain reaction (PCR) test. However, this method is not error free and sometimes gives false results [2]. Another difficulty comes from the fact that some people, despite being infected with the SARS-CoV-2 virus, do not develop symptoms of the disease [3] and are not referred for PCR testing. This can cause problems with the correct diagnosis of the disease. Despite its latency, the disease can cause serious changes in the lungs. In these cases, the diagnosis is possible on the basis of an X-ray of the lungs. For this reason, machine learning methods have been used to detect COVID-19 infections on X-ray images. Methods based on machine learning (ML) turned out to be effective and useful during the analysis and assessment of the impact of diseases (e.g., COVID-19 or pneumonia) on X-ray images of the lungs [4,5,6]. The major contributions of this paper are as follows:

- We propose a novel approach to chest X-ray image analysis in order to diagnose COVID-19 using an original CNN-based features extraction method.
- We obtained a new dataset containing samples from confirmed COVID-19 cases as well as from uninfected patients. The infection status of both groups was confirmed by a PCR test. We performed an augmentation in order to increase the dataset’s size.
- We implemented the proposed features extraction for different classifiers, obtaining promising results.

Further parts of this paper are constructed in the following manner: (a) a brief review of the state-of-the-art is presented in Section 2; (b) in Section 3, we describe the dataset, augmentation process and the proposed approach to the classification; (c) in Section 4, we present the obtained results; (d) in Section 5 (the Discussion) we compare our results with other state-of-the-art approaches, we pull out some conclusions, and we present perspectives for future work on this topic.

## 2. Related Work

To combat the challenges posed by the pandemic to the healthcare service, Khan et al. [7] proposed a deep-learning-based method of accurate and quick diagnosis of COVID-19 using X-ray images. They proposed a method consisting of two novel deep learning frameworks: Deep Hybrid Learning (DHL) and Deep Boosted Hybrid Learning (DBHL). The use of both of these frameworks led to an improvement in their COVID-19 diagnostic methods. The result was a model capable of identifying COVID-19 in X-ray images with over 98% accuracy on a previously unseen dataset. This method has been shown to be effective in reducing both false positives and false negatives and has proven to be a useful supportive tool for radiologists.

Tahir et al. [8] proposed a model based on a convolutional neural network (CNN) capable of lung segmentation and localization of specific changes caused by COVID-19. The dataset used consisted of nearly 34,000 X-ray images, including lung images of people with COVID-19 and pneumonia and of healthy people. An important element of the study was the appropriate marking of photos by specialists. This method recognized COVID-19 and its effects on the lung image with sensitivity and specificity values over 99%.

In [9], Brunese et al. used transfer learning to create a model capable of detecting COVID-19 changes in X-ray images of the lungs. This model is applicable (1) to the classification of healthy people and patients with changes in lung X-ray images; (2) to distinguish between COVID-19 and other lung diseases; and (3) to distinguish lung lesions caused by the SARS-CoV-2 infection. This model was based on the VGG-16 (16-layered convolutional neural network) and underwent transfer learning. This study included 6523 X-ray images from healthy individuals, patients with various lung diseases and patients with COVID-19. The model trained on this dataset achieved a sensitivity equal to 0.96 and a specificity of 0.98 (accuracy of 0.96) for distinguishing between healthy individuals and patients with lung diseases, and a sensitivity of 0.87 and a specificity equal to 0.94 (accuracy of 0.98) for distinguishing lung diseases from COVID-19. The image analysis process itself is extremely fast and takes only about 2.5 s. The data presented by the authors indicate that the developed model achieves good and reliable results.

Chakraborty et al. [10] presented a COVID-19 detection method based on a Deep Learning Method (DLM) using X-ray images. The authors used different architectures of deep neural networks in order to achieve optimal results. They combined several pre-trained models such as ResNet18, AlexNet, DenseNet, VGG16, etc. This approach showed to be effective and cost significantly less than standard laboratory diagnostic methods. The dataset consisted of 10,040 chest X-ray images, which included a normal/healthy population, COVID-19 patients and patients with pneumonia. The presented model was highly accurate (96.43%) and sensitive (93.68%). This work showed the high usefulness of ML models for determining changes in X-ray images, which can facilitate the work of radiologists who, as a result of this quick method, can refer patients directly to treatment.

Civit-Masot et al. [11] noted that traditional tests to identify SARS-CoV-2 infection are invasive and time consuming. Imaging, on the other hand, is a useful method for assessing disease symptoms. Due to the limited number of trained medical doctors who can reliably assess X-ray images, it is necessary to invent ways to facilitate this type of assessment. The authors used the VGG-16-based Deep Learning model to identify pneumonia and COVID-19. The presented results indicate high accuracy (close to 100%) and specificity of the model, which qualify it as an effective screening test.

It is also worth mentioning that ML-based methods can support not only radiology specialists. In [12], we can see the transfer learning approach to discovering the impact of the stringency index on the number of deaths caused by the SARS-Cov-2 virus. As presented in [13], ML can be also implemented in order to predict the COVID-19 diagnosis based on symptoms. Statistical analyses revealed that the most frequent and significant predictive symptoms are fevers (41.1%), coughs (30.3%), lung infections (13.1%) and runny noses (8.43%). A total of 54.4% of people examined did not develop any symptoms that could be used for diagnosis. Moreover, ML can also be a useful tool in vaccine discovering, as presented in [14].

Recently, numerous ML-based approaches for rapid diagnostics have been published. In addition, they have gathered increasingly more attention from some government and international agencies. For example, the European Commission published a White Paper entitled ‘On Artificial Intelligence—A European approach to excellence and trust [15]. Seven key requirements were identified and are described in the document:

- Human agencies and oversight;
- Technical robustness and safety;
- Privacy and data governance;
- Transparency;
- Diversity, non-discrimination and fairness;
- Societal and environmental well-being;
- Accountability.

The following statement in the EC publication is worth noting: AI can and should itself critically examine resource usage and energy consumption and should be trained to make choices that are positive for the environment. It follows from the above citation that it is extremely important to focus on providing solutions that are not only cost and time effective but also that spare energy used for computations. This kind of approach to AI has become introduced as the ‘GreenAI’ [16] and has gathered increasingly more attention recently [17]. Research working on the GreenAI have also proposed some novel metrics [18]. As a result of this metric, researchers can compare not only the accuracy and precision of the proposed method, but also its sustainability and eco-friendliness.

## 3. Materials and Methods

**Figure 1:**
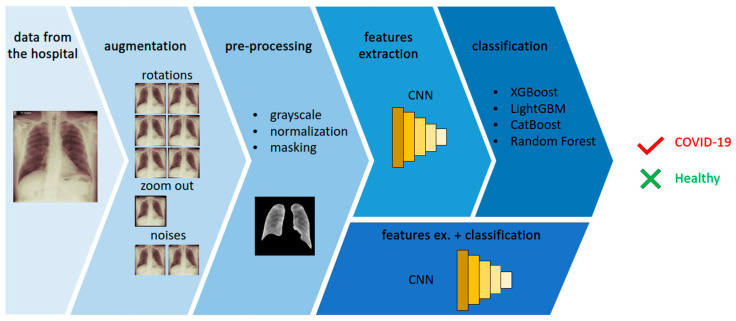
The following steps of processing in the proposed method: data acquisition, data augmentation, sample pre-processing, features extraction and binary classification of COVID-19 as positive or negative (healthy).

The general overview of the proposed method is presented in Figure 1. The image presents an example of the data obtained from the hospital and some consequential steps: the augmentation process and pre-processing of the sample. In Figure 1, the features extraction and classification steps are presented. Finally, the proposed method gives the answer of “*true*” for the COVID-19-positive sample and “*false*” for the healthy sample. Each step of the process is described in detail in this section. All experiments were carried out with the use of Python 3.7 and the TensorFlow platform. Among others, we used the following libraries: scikit-learn, Xgboost, Lightgbm and Catboost.

### 3.1. Dataset

**Figure 2:**
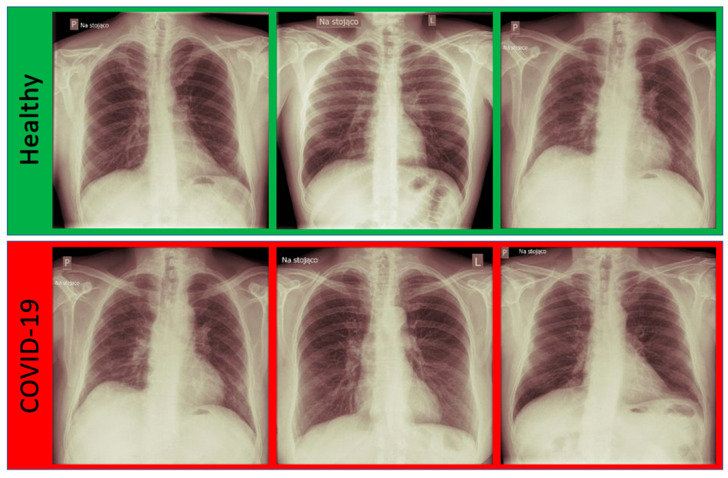
The exemplary images from the dataset divided into two classes: Healthy and COVID-19 confirmed by a PCR test.

In this research, anonymized real data were used. The data were obtained from Antoni Jurasz University Hospital No. 1 in Bydgoszcz, Department of Radiology and Imaging Diagnostics. A total of 60 chest X-ray images were obtained; 30 were from healthy individuals, and 30 had COVID-19 confirmed by a PCR test. The images were provided in the DICOM format. The images were in a raw form, without masks. Some samples from the dataset are presented in Figure 2.

### 3.2. Data Augmentation

In order to perform training, the dataset was divided into 3 disjointed subsets: the training set (80%), validation set (10%) and testing set (10%). Unfortunately, the quantity of samples was not enough to use any ML technique. Thus, we decided to use augmentation for increasing the size of the training dataset. As a result of the augmentation, 10 samples from one single image were obtained. The initial proper balance in the dataset was unchanged, and as a result, the dataset was still well balanced. The following methods for augmentation were implemented:

- rotations—1°, 2° and 3° both clockwise and anti-clockwise;
- noises—a random Gaussian noise and a salt and pepper noise were added;
- zooming out—the image was resized to obtain 95% of its original size.

### 3.3. Data Pre-Processing

First of all, the samples were moved to grayscale images and were normalized. The goal of normalization was to improve the quality of the images, e.g., by enhancing the contrast, as described in [19]. The data obtained from the hospital were not masked. Thus, the essential step of processing was to provide proper masks to help in selecting the region of interest. The goal of this step was to prevent the ML-based model from learning information that is useless from the point of view of COVID-19 diagnostics, such as images of a collar bone or a stomach. Hand crafting masks would be time consuming, and it would require the involvement of a specialist. On the other hand, proposing a novel method of segmentation can be treated as a separate scientific problem, as presented in [20,21]. Therefore, it was decided to use the pretrained model, which is widely available and very powerful in masking X-ray images [22].

### 3.4. ML-Based Methods

ML-based methods were used in two steps of processing, namely features extraction and classification. As a baseline, a convolutional neural network (CNN) was used for both steps. Then, almost the same CNN architecture was used solely for extracting features, since it was reported as very promising and efficient [23,24].

**Figure 3:**
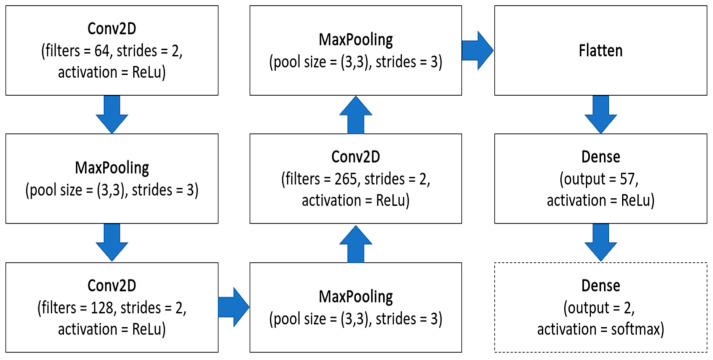
The architecture of the CNN used in the research. In dashed lines, the added Dense network was in solely a CNN-based approach.

The features extraction step can be an essential one for the whole image processing system. It can reduce the complexity of the problem, and consequently, it can make the proposed approach more efficient, require less computing time and, therefore, more eco-friendly. The general schema of the CNN is presented in Figure 3. The input in this architecture was grayscale images with sizes of 512 × 512 pixels. Then, three pairs of convolutional layers and max pooling layers were used. Each of them was responsible for performing operations between the filters and the input of each corresponding layer. The convolutional layers consisted of 64, 128 and 256 filters, respectively. On each layer, an ReLU activation function was used to implement and perform nonlinear transformations. Then, the flattened layer and the dense layer were used. The proposed neural networks against the dense layer neuron quantity were examined. The values, with a range of [5,200], were tested. Involving the validation subset, it was observed that the most promising was using 57 features. This features extraction type was qualified for further research and development. Then, numerous classifiers were examined: XGBoost [25], Random Forest [26], LightGBM [27] and CatBoost [28]. It was decided to use these classifiers due to some reasons. First of all, they are tree-based algorithms, and they perform very well in binary classification problems. Secondly, they have been already used in similar applications, as presented in [29,30,31], providing promising results.

The approach based on solely CNN both for features extraction and for classification had one change in the architecture. In this case, the next dense layer with the softmax activation function (presented in Figure 3 in dashed line) was added. It enabled the binary classification of COVID-19 positive or negative. This approach was trained, validated and tested using the above-mentioned training, validation and testing datasets, respectively. The training parameters were: 50 epochs, a learning rate equal to 0.00001 and a loss function set to SparseCategoricalCrossentropy.

## 4. Results

Since the hospital data represent two classes (COVID-19 positive and healthy), the problem of the disease diagnostics can be treated as a binary classification. In this research, the confusion matrices were used in order to evaluate and compare the ML-based methods. Four measures were defined, as follows:

- TP—true positives—COVID-19-infected patients classified as sick;
- FP—false positives—healthy patient images classified as COVID-19 infected;
- FN—false negatives—COVID-19-infected patients classified as healthy;
- TN—true negatives—healthy patients classified as healthy.

Each model in the research was evaluated using accuracy (Equation (1)), precision (Equation (2)), recall (Equation (3)) and F1-score (Equation (4)), which use the above-mentioned measures of TP, FP, FN and TN.

(1) $$\mathrm{Acc}=\frac{\mathrm{TP}+\mathrm{TN}}{\mathrm{TP}+\mathrm{TN}+\mathrm{FP}+\mathrm{FN}},$$

(2) $$\mathrm{Precision}=\frac{\mathrm{TP}}{\mathrm{TP}+\mathrm{FP}},$$

(3) $$\mathrm{Recall}=\frac{\mathrm{TP}}{\mathrm{TP}+\mathrm{FN}},$$

(4) $$F1\text{-}\mathrm{score}=\frac{2\cdot \mathrm{Precision}\cdot \mathrm{Recall}}{\mathrm{Precision}+\mathrm{Recall}},$$

All experiments were performed using a Tesla with GPU support. As a result of its enormous computing power, low price, relatively low demand for electricity and the CUDA environment support, Tesla systems have become an attractive alternative to traditional high-power computing systems, such as CPU clusters and supercomputers. This kind of device can be extremely helpful in image processing and also in medicine diagnostics.

The obtained results from all the experiments are provided in Table 1. All the evaluated metrics are given: accuracy, precision, recall and F1-score. The approach using the CNN both for features extraction and for classification provided the less promising results. Two examined classifiers provided the highest results: XGBoost and LightGBM, with accuracy = 1.0, precision = 1.0, recall = 1.0 and F1-score = 1.0. For selecting the optimal classifier for the presented solution, the computational time for both classifiers, namely the training time and prediction time for a single image, was compared. It was decided to use this parameter for optimization because the goal was to provide a light, sustainable and eco-friendly solution. For XGBoost, the average training time was equal to 242 ms, and the prediction time for a single image was above 11ms. For LightGBM, those times were 132 ms and less than 2 ms, respectively. That is why LightGBM is marked in bold in Table 1.

One could ask ‘what makes LightGBM faster than XGBoost?’. The following are the features of LightGBM that affect its effectiveness and the mathematics (Equation (5)) behind LightGBM that allow one to understand the answer to this question [32,33].

(5) $$V_{j}^{}\left(d\right)=\frac{1}{n}$$

where $A_{l}=\left\{x_{i}\in A:x_{ij}\leq d\right\}$, $A_{r}=\left\{x_{i}\in A:x_{ij}>d\right\}$, $B_{l}=\left\{x_{i}\in B:x_{ij}\leq d\right\}$, $B_{r}=\left\{x_{i}\in B:x_{ij}>d\right\}$, *d* is the point in the data where the split is calculated to find the optimal gain in variance and the coefficient $\frac{1-a}{b}$ is used to normalize the sum of the gradients over B back to the size of $A^{C}$.

LightGBM produces trees and finds the leaves with the greatest variance to perform division with the use of leaf-sage techniques. LightGBM achieves the optimal number of leaves in the trees and uses the minimum amount of data in the tree.

## 5. Discussion

ML methods can be valuable tool for COVID-19 diagnosis. ML-based methods cannot replace an experienced medical doctor in the final diagnosis, but they help significantly in the process, relieving the burden on health care and improving the diagnostic process. Screening with X-ray images is less expensive and faster than PCR testing [34]. This is one of the reasons why it is worth developing ML-based techniques to assist specialists in diagnostics.

In [35], the authors paid particular attention to the explainability AI (xAI), as it is essential in clinical applications. Explainable approaches increase the confidence and trust of the medical community in AI-based methods. They noted that X-ray imaging was not a method of choice when diagnosing COVID-19. However, the changes visible in the X-ray images of the lungs allow for the detection of pathological changes at an early stage of their development. For this reason, the authors indicated the usefulness of models supporting radiologists in their work and improving the decision-making process. The dataset of X-ray images used by the authors contained nearly 900 X-ray images of both COVID-19 patients and healthy patients, which was a significantly bigger dataset than that presented in this paper. Therefore, it could learn a wider range of differences between the images. In this work, the authors used pre-trained networks (ResNet-18 and DenseNet-121) to perform image classification with the best AUC score of 0.81. The sensitivity and specificity results obtained by authors were significantly lower than ours, however, which may be caused by dataset size differences. The model proposed in this research is fast, efficient and does not require high computing power; thus, it can be used in ordinary computers in hospital laboratories. The presented model obtained satisfactory results of evaluation metrics, which confirm its accuracy. These results are comparable and potentially better than those reported in the state-of-the-art review (Section 2). Some detailed results provided in the literature are presented in Table 2. However, our concern remains on the small number of original images that formed the basis of the database used. We believe that the method of data augmentation used may introduce bias; however, with such a small amount of data, this step was necessary. We believe that this is an aspect that could be improved in the course of further cooperation with hospitals that would provide more learning data.

Table 2 presents numerous approaches providing comparable results. It is essential to mention that some of them are based on very complex architectures, use an extremely big number of training parameters, need excessive computational power and require long training. However, we decided to use fewer than 60 features and a light, fast classifier in our approach. Thus, the approach proposed in this paper is lightweight, efficient and fast. In the literature, we can observe very complex and resource-consuming approaches (such as COVID-Net, as proposed in [37], or ResNet, as implemented in [38]). It is worth emphasizing that the proposed solution is lighter but still equally or more efficient. Unfortunately, it is very difficult to compare the eco-friendliness of different computing-based diagnostic approaches, as it is not customary to provide such information in scientific publications. Hopefully, in the near future, the sustainability of the proposed methods will become more significant for researchers and editors.

We are also aware that there are some future improvements required for the presented model. It is necessary to validate the model on a larger, different dataset. X-ray images made with the use of various equipment exhibit different features, which may be an obstacle to the universality of the presented model. It is worth checking how the model performs on a new set of data from the same hospital and, alternatively, on a set from a different source. Likely, the additional pre-processing can help to make all samples uniform. Another potential extension of this work is providing the xAI. Its main aim is not only giving the classification but also providing an explanation of why such a decision was made by a ML-based method. Implementing the xAI can allow radiologist doctors to evaluate the model and verify whether it makes the decisions based on real COVID-19 lesions.

## Figures and Tables

**Table 1:** Obtained results: accuracy, precision, recall and F1-score for all experiments.

F. Extractor	Classifier	Accuracy	Precision	Recall	F1-Score
CNN	CNN	0.86	0.75	1.00	0.86
CNN	XGBoost	1.00	1.00	1.00	1.00
CNN	Random Forest	0.91	0.86	1.00	0.92
CNN	LightGBM	1.00	1.00	1.00	1.00
CNN	CatBoost	0.91	0.86	1.00	0.92

**Table 2:** Results compared to other state-of-the-art methods, namely accuracy, precision, recall, F1-score and AUC. The results not provided by the authors are marked with ‘-’.

Authors	Method	Acc.	Prec.	Rec.	F1	AUC
Rajagopal [27]	CNN + SVM	0.95	0.95	0.95	0.96	-
Júnior et al. [30]	VGG19 + XGBoost	0.99	0.99	0.99	0.99	-
Nasari et al. [29]	DenseNet169 + XGBoost	0.98	0.98	0.92	0.97	-
Ezzoddin et al. [36]	DenseNet169 + LightGBM	0.99	0.99	1.00	0.99	-
Laeli et al. [28]	CNN + RF	0.99	-	-	-	0.99
Proposed	CNN + LightGBM	1.00	1.00	1.00	1.00	1.00

## Data Availability

The data used in the research in a raw, anonymized form are available at https://github.com/UTP-WTIiE/Xray_data.git (accessed on 2 August 2022).
